# Atrial fibrillation or flutter in patients undergoing stem cell transplantation, in-hospital and post-discharge outcomes in a large nationwide sample across the United States

**DOI:** 10.1186/s40959-025-00346-1

**Published:** 2025-07-03

**Authors:** Raja Zaghlol, Elena Deych, Nina Manian, Ahmed Altibi, Joshua D. Mitchell

**Affiliations:** 1 Division of Cardiovascular Disease, Cardio-Oncology Section, Washington University School of Medicine, Campus Box 8086, 660 South Euclid Ave., St. Louis, MO 63110 USA; 2 Division of Internal Medicine, Washington University School of Medicine, St. Louis, MO USA; 3 Division of Cardiovascular Disease, Electrophysiology Section, Yale University, New Haven, CT USA

**Keywords:** Atrial arrhythmias, Atrial fibrillation, Atrial flutter, Stem cell transplantation, Bone marrow

## Abstract

**Background:**

Stem Cell Transplantation (SCT) is a cornerstone therapy in managing several malignant and benign hematological conditions. Atrial fibrillation/atrial flutter (AF) are commonly encountered in patients receiving SCT. There is a paucity of large-scale data on the prevalence of AF and their effect on outcomes following SCT.

**Methods:**

The United States National Readmission Database (NRD) was used to identify hospitalized patients who underwent SCT. Baseline demographics, comorbidities, the presence or absence of AF, the indication, and type of SCT were identified using diagnostic and procedural International Classification of Diseases 10th Edition (ICD-10) codes. Patients with AF were compared to those without AF for differences in baseline characteristics, in-hospital mortality, cardiovascular (CV) complications, length and cost of hospitalization, and post-discharge 90-day readmissions and mortality.

**Results:**

Between January 2016 and September 2020 there were 59,284 weighted admissions for SCT, of which 5797 (9.8%) patients had AF. Patients in the AF group were more likely to be older males with an increased burden of baseline comorbidities compared to the no-AF group ((64 [9] vs. 56 [14] years, *p *< 0.001) and (3893 [67%] vs. 30,886 [58%] males, *p* < 0.001) respectively). Adjusted for differences in baseline demographics, comorbidities, indication and type of SCT, patients with AF had higher in-hospital mortality (adjusted odds ratio (AOR) 3.65 [3.02–4.41]) and adverse events including cardiac complications [composite of acute heart failure, acute myocardial infarction, cardiogenic shock, and cardiac arrest] (AOR 4.92 [4.22–5.75]), bleeding (AOR 1.32 [1.15–1.53]), and respiratory failure (AOR 3.40 [2.97–3.90]) compared to patients without AF. Additionally, the AF group had longer hospitalizations (21 [16–27] vs. 19 [15–25] days, *p* < 0.001) with higher cost ($268,031 [$170,957-$455739] vs. $250,178 [$153,680-$415239], p < 0.001) compared to the no-AF group. Among survivors to hospital discharge, patients with AF also had higher adjusted 90-day all-cause inpatient mortality (adjusted hazard ratio (AHR) 1.54 [1.19–1.99], *p* = 0.001), all-cause readmissions (AHR 1.15 [1.07–1.24],* p *< 0.001), and CV readmissions (AHR 2.29 [1.85–2.82], *p* < 0.001).

**Conclusions:**

In a large national cohort of SCT recipients, AF were common and independently associated with increased in-hospital mortality and CV adverse events, along with increased 90-day mortality and readmissions among survivors to hospital discharge.

**Supplementary Information:**

The online version contains supplementary material available at 10.1186/s40959-025-00346-1.

## Background

Stem cell transplantation (SCT) is a cornerstone therapy for several malignant and benign hematologic conditions, with around 23,000 transplantations performed in the United States (US) annually [1]. As transplantation strategies improve, the number of SCT survivors is growing [2, 3] with an estimated doubling from around 242,000 in 2020 to over 500,000 in 2030 [4]. These SCT survivors are at an increased risk of cardiovascular (CV) adverse events and mortality exceeding that of the general population [5].

Atrial fibrillation or atrial flutter (AF) are the most common arrhythmias in the general population, increasing in prevalence with age to affect over a third of patients above the age of 80 [6, 7]. AF pose a significant burden on the healthcare system as they are associated with increased risk of mortality, strokes, and heart failure (HF) [6, 7].

AF are commonly encountered in patients with cancers, particularly recipients of SCT [8]. Increased prevalence of CV risk factors, arrhythmogenic chemotherapies and conditioning regimens received around SCT, a hyperinflammatory state associated with SCT, infections, and physical stressors are among factors that may explain this association [9]. Small single-centered studies reported increased risk of mortality and adverse events in SCT patients with AF compared to those without AF, however, large nationwide real-world data are still lacking [9, 10]. There are also a paucity of data regarding the effect of AF on healthcare resource utilization such as hospitalization costs and readmission rates following SCT. We aim to leverage data from the large, United States National Re-Admission Database (NRD) to investigate the association between AF and clinical outcomes, CV events, and resource utilization during and following the admission for SCT.

## Methods

The National Readmission Database (NRD) is part of a family of databases developed for the Healthcare Cost and Utilization Project (HCUP). It provides all-payer inpatient data for admissions and re-admissions in the US for facilities participating in the HCUP. As of 2020 the NRD contains data from approximately 17 million unweighted and 32 million weighted annual discharges [11].

### Cohort selection

International Classification of Diseases 10 th Edition Procedural Coding System (ICD-10-PCS) codes were used to identify hospitalized patients ≥ 18-year-old who underwent SCT between January 1 st, 2016, and September 30 th, 2020. Patients with more than one admission for SCT in the same year were excluded. Hospital type (teaching vs. non-teaching) and size, along with patients’ baseline sociodemographic characteristics and medical comorbidities were extracted from the NRD baseline data file and ICD-10 diagnostic codes. The indication for SCT was identified using ICD-10 diagnostic codes for cancers commonly treated with SCT including multiple myeloma, acute lymphocytic leukemia (ALL), acute myeloid leukemia (AML), Hodgkin lymphoma, non-Hodgkin lymphoma, and myelodysplastic syndrome (MDS). When none of those diagnostic codes were identified, the indication for SCT was labelled as “Other.” Patients were further categorized by type of SCT (autologous or allogenic) and source of SCT (hematopoietic, bone marrow, or cord) according to their ICD-10- procedural codes. The previously validated Charlson Comorbidity Index was used to capture and account for differences in baseline medical comorbidities [12, 13]. Patients were divided into two groups (AF group and no-AF group) based on the presence or absence of AF diagnosis during the SCT hospitalization (including pre-existing and de-novo AF). The diagnosis of AF was defined by the presence of ICD-10 diagnostic code I48 in any diagnostic position during the SCT admission. Patients with AF were compared to patients without AF for differences in baseline characteristics and clinical outcomes. Weighted national estimates were used for the analysis using standard NRD weighting methods [14]. The diagnostic and procedural codes used to create the cohort are shown in Supplemental Table 1.

**Table 1:** baseline socio-demographics and medical comorbidities

Baseline characteristic	All Patients *(n* = 59,284)	No AF (*n* = 53,487)	AF (*n* = 5797)	*p*-value	Absolute Standardized Difference
**Demographics**					
Age, mean (SD)	56 (13)	56 (14)	64 (9)	< 0.001	0.70
Male gender, n (%)	34,778 (59%)	30,886 (58%)	3,893 (67%)	< 0.001	0.19
Primary payer, n (%)				< 0.001	0.45
Medicaid	6,238 (11%)	6,008 (11%)	231 (4.0%)		
Medicare	17,683 (30%)	14,943 (28%)	2,739 (47%)		
Private	32,721 (55%)	30,098 (56%)	2,623 (45%)		
Other	2,571 (4.3%)	2,372 (4.4%)	200 (3.4%)		
Zip Code income quartile, n (%)				0.071	0.07
1	11,928 (20%)	10,885 (20%)	1,042 (18%)		
2	15,179 (26%)	13,650 (26%)	1,529 (26%)		
3	16,098 (27%)	14,432 (27%)	1,666 (29%)		
4	15,373 (26%)	13,876 (26%)	1,497 (26%)		
Hospital bed size, n (%)				0.006	0.08
Small	3,865 (6.5%)	3,513 (6.6%)	352 (6.1%)		
Medium	5,659 (9.5%)	5,213 (9.7%)	446 (7.7%)		
Large	49,759 (84%)	44,761 (84%)	4,999 (86%)		
Teaching hospital, n (%)	58,300 (98%)	52,574 (98%)	5,725 (99%)	0.088	0.04
**Type of SCT, n (%)**					
Allogenic	20,041 (34%)	18,260 (34%)	1,781 (31%)	0.002	0.07
Autologous	39,243 (66%)	35,227 (66%)	4,016 (69%)		
**Source of stem cells, n (%)**					
Bone marrow	2,206 (3.7%)	2,015 (3.8%)	191 (3.3%)	0.6	0.03
Cord	846 (1.4%)	758 (1.4%)	88 (1.5%)		
Hematopoietic	56,232 (95%)	50,714 (95%)	5,518 (95%)		
**Indication for SCT, n (%)**					
ALL	2,693 (4.5%)	2,544 (4.8%)	150 (2.6%)	< 0.001	0.24
AML	7,521 (13%)	6,859 (13%)	662 (11%)		
Hodgkin’s lymphoma	2,834 (4.8%)	2,709 (5.1%)	125 (2.2%)		
MDS	3,197 (5.4%)	2,830 (5.3%)	367 (6.3%)		
Multiple myeloma	24,499 (41%)	21,995 (41%)	2,504 (43%)		
Non-Hodgkin’s lymphoma	11,736 (20%)	10,324 (19%)	1,412 (24%)		
Other	6,804 (11%)	6,225 (12%)	578 (10.0%)		
**Comorbidity, n (%)**					
Obesity	7,296 (12%)	6,404 (12%)	892 (15%)	< 0.001	0.10
Hypertension	28,537 (48%)	24,968 (47%)	3,569 (62%)	< 0.001	0.30
Diabetes	9,145 (15%)	8,000 (15%)	1,145 (20%)	< 0.001	0.13
Coronary Artery Disease	4,266 (7.3%)	3,382 (6.3%)	884 (15%)	< 0.001	0.29
COPD	2,279 (3.8%)	1,888 (3.5%)	391 (6.7%)	< 0.001	0.15
CKD stage 3 or more	5,552 (9.4%)	4,555 (8.5%)	997 (17%)	< 0.001	0.26
Prior CABG	627 (1.1%)	486 (0.9%)	141 (2.4%)	< 0.001	0.12
Hyperthyroidism	247 (0.4%)	208 (0.4%)	38 (0.7%)	0.021	0.04
Prior Stroke/TIA	1,293 (2.2%)	1,098 (2.1%)	196 (3.4%)	< 0.001	0.08
Peripheral vascular disease	533 (0.9%)	454 (0.8%)	80 (1.4%)	0.012	0.05
Heart failure	2,117 (3.6%)	1,520 (2.8%)	596 (10%)	< 0.001	0.30
Sleep apnea	4,100 (6.9%)	3,412 (6.4%)	688 (12%)	< 0.001	0.19
Alcohol use disorder	448 (0.7%)	401 (0.8%)	47 (0.8%)	0.8	0.01
**Charlson Comorbidity Index, median (IQR)**	2.00 (2.00, 3.00)	2.00 (2.00, 3.00)	3.00 (2.00, 4.00)	< 0.001	0.30

Baseline characteristics including socio-demographics and medical comorbidities were identified using ICD-10 diagnostic and procedural codes of claims and NRD baseline data at the time of the index hospitalization for SCT. Patients were divided into two groups (AF and no AF) by the presence or absence of AF diagnostic codes at the time of SCT. The Charlson Comorbidity Index was calculated using previously validated and published methods. Categorical variables are shown as frequency (percentage) while continuous variables are reported as mean (SD) or median (IQR) based on data normality. Between groups comparison was performed using Chi-squared testing for categorical variables and T-test or Wilcoxon test for continuous variable guided by normality testing. While p-values are reported, due to large sample size the differences between the groups were mainly assessed using standardized differences. An absolute difference of greater than 0.1 was considered clinically significant

*AF* atrial fibrillation/atrial flutter, *SD* standard deviation, *SCT* stem cell transplantation, *ALL* acute lymphoblastic leukemia, *AML* acute myelogenous leukemia, *MDS* myelodysplastic syndrome, *COPD* chronic obstructive pulmonary disease, *CKD* chronic kidney disease, *CABG* coronary artery bypass grafting, *TIA* transient ischemic attack, *IQR* interquartile range

### Clinical outcomes during index admission and follow-up

The index admission (IA) was defined as the hospitalization in which the patient underwent SCT. Since the NRD structure does not allow follow-up after the end of a calendar year into the next calendar year, patients were only included if their IA occurred between January 1 st and September 30 th of each year to allow for at least 90-day follow-up. (Patients admitted between October 1 st and December 31 st of each year were excluded).

During the IA, patients in the AF group were compared to those in the no-AF group for differences in inpatient mortality, length of stay, cost of hospitalization, and in-hospital complications including acute HF, acute myocardial infarction (MI), cardiogenic shock, cardiac arrest, acute kidney injury, bleeding complications, respiratory complications, and arterial thromboembolic events using previously published ICD-10 codes [15] (Supplemental Table 2). For adjusted analyses, in-hospital events were grouped into a) cardiac complications, including a composite of acute HF, acute MI, cardiogenic shock, and cardiac arrest, b) bleeding complications, including a composite of gastrointestinal (GI), genitourinary, nasopulmonary, intracranial, and unspecified bleeding, and c) respiratory complications, including a composite of acute respiratory failure, non-invasive positive pressure ventilation, and mechanical ventilation.

**Table 2:** Adjusted in-hospital outcomes in patients admitted for SCT with AF

Outcome	Adjusted OR (95% CI) AF vs. No AF	*P*-value
Mortality	**3.65 (3.02–4.41)**	** < 0.0001**
Cardiac Complications^a^	**4.83 (4.14–5.63)**	** < 0.0001**
Bleeding Events^b^	**1.32 (1.15–1.53)**	**0.0001**
Respiratory Failure^c^	**3.4 (2.97–3.9)**	** < 0.0001**
Length of stay	**IRR of 1.12 (1.1–1.15)**	** < 0.0001**

Multivariable logistic regression models were constructed to adjust in-hospital outcomes for the following differences in baseline characteristics at the time of SCT: age, gender, Charlson Comorbidity Index, primary payer, type, and indication of SCT. The adjusted odds ratio (or incidence rate ratio) compares the likelihood of the outcome in patients with AF compared to those without AF during their SCT hospitalization

*AF* atrial fibrillation/atrial flutter, *OR* odds ratio, *IRR* incidence rate ratio

^a^ Composite of acute heart failure, acute myocardial infarction, cardiogenic shock, and cardiac arrest

^b^ Composite of gastrointestinal, genitourinary, nasopulmonary, intracranial, and other bleeding events

^c^ Composite of acute respiratory failure, non-invasive positive pressure ventilation, and mechanical ventilation

Patients alive at discharge were followed until death or censoring at the end of the calendar year. The primary post-discharge outcomes were 90-day all-cause inpatient mortality and all-cause readmissions. Secondary outcomes were 180-day all-cause inpatient mortality and all-cause readmissions, along with CV readmissions until the end of the calendar year. CV readmissions were defined as a hospital readmission with a CV diagnosis (I00-I99 ICD-10 diagnostic code) in the primary diagnostic position. Etiologies of CV readmissions were further grouped into the most common etiologies including AF, cerebrovascular accident (CVAs) (hemorrhagic or ischemic), acute HF, hypertensive complications, hypotension, ischemic heart disease, pericardial disease, and pulmonary embolism. Time between discharge after the IA until death or readmission was calculated by standard NRD methods using the database *DaysToEvent* element.

Outcomes during IA and follow-up were statistically adjusted to account for differences in baseline characteristics at the time of SCT including age, gender, Charlson Comorbidity Index, and primary payer along with differences in the type and indication of SCT. Given the association between AF and other in-hospital complications that could confound the outcomes such as sepsis and respiratory failure, and the lack of temporal associations in administrative data (distinguishing if AF occurred before or after sepsis or respiratory failure), we also performed sensitivity analyses excluding patients who had sepsis or respiratory failure during IA.

Patients were evaluated for new and recurrent AF during follow up by identifying readmissions for a primary diagnosis of AF in patients with preexisting AF during IA, and readmissions with AF in any diagnostic position among those without AF in the IA.

### Statistical analysis

All statistical analysis used survey weights adjusted methods. For univariate analysis, continuous variables were reported as means (standard deviation [SD]) or median (interquartile range [IQR]), as appropriate based on data distribution. Categorical variables were reported as frequency (proportion). Although p-values using t-test, Wilcoxon test, or Chi-squared tests were calculated, due to large sample size the differences between the groups were mainly assessed using standardized differences. An absolute difference of greater than 0.1 was considered clinically significant.

Time to event outcomes were compared between groups univariately using Kaplan–Meier curves with log-rank tests (adjusting for survey weights). Likewise, multivariable Cox proportional models were adjusted for survey weights and proportionality assumptions were tested and confirmed for all models. All analyses were performed using R version 4.3.2.

## Results

**Fig. 1:**
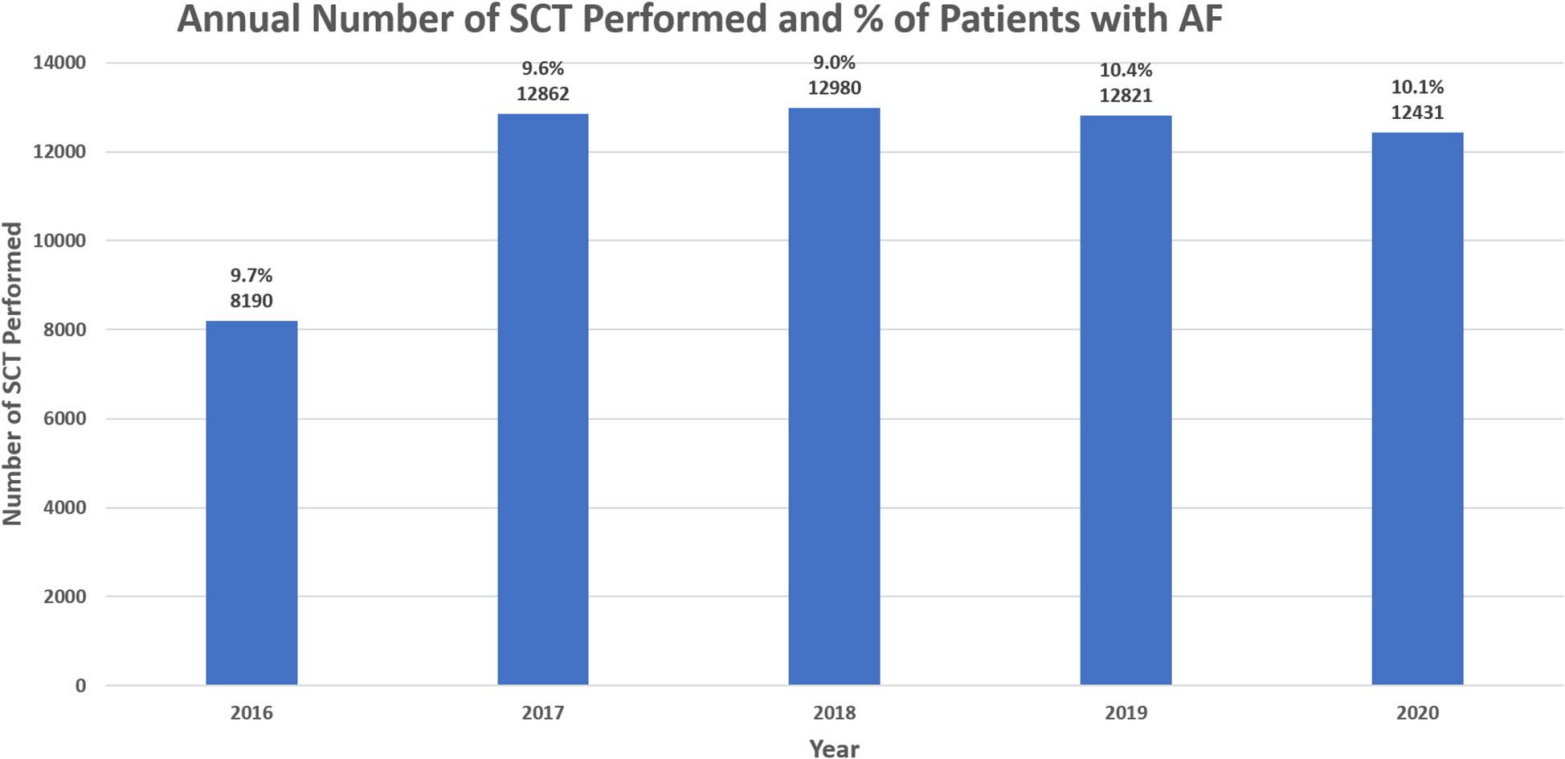
Annual Trends for SCT and patients with AF. SCT: stem cell transplantation; AF: atrial fibrillation/atrial flutter

Between January 2016 and September 2020 there were 59,284 weighted admissions for SCT, of which 5797 (9.8%) patients had AF. The number of SCT performed and the percentage of patients with atrial AF was relatively stable over the study period (Fig. 1). Patients with AF were older (64 [SD of 9] vs. 56 [14] years, standardized difference 0.70) with more males (3893 [67%] vs. 30,886 [58%], standardized difference 0.19) compared to patients without AF. Multiple myeloma was the most common indication for SCT in both groups, accounting for about 42% of the cases. Patients in the AF group also had more baseline CV risk factors and comorbidities compared to the no-AF group (Table 1).

### Outcomes during index admission

**Fig. 2:**
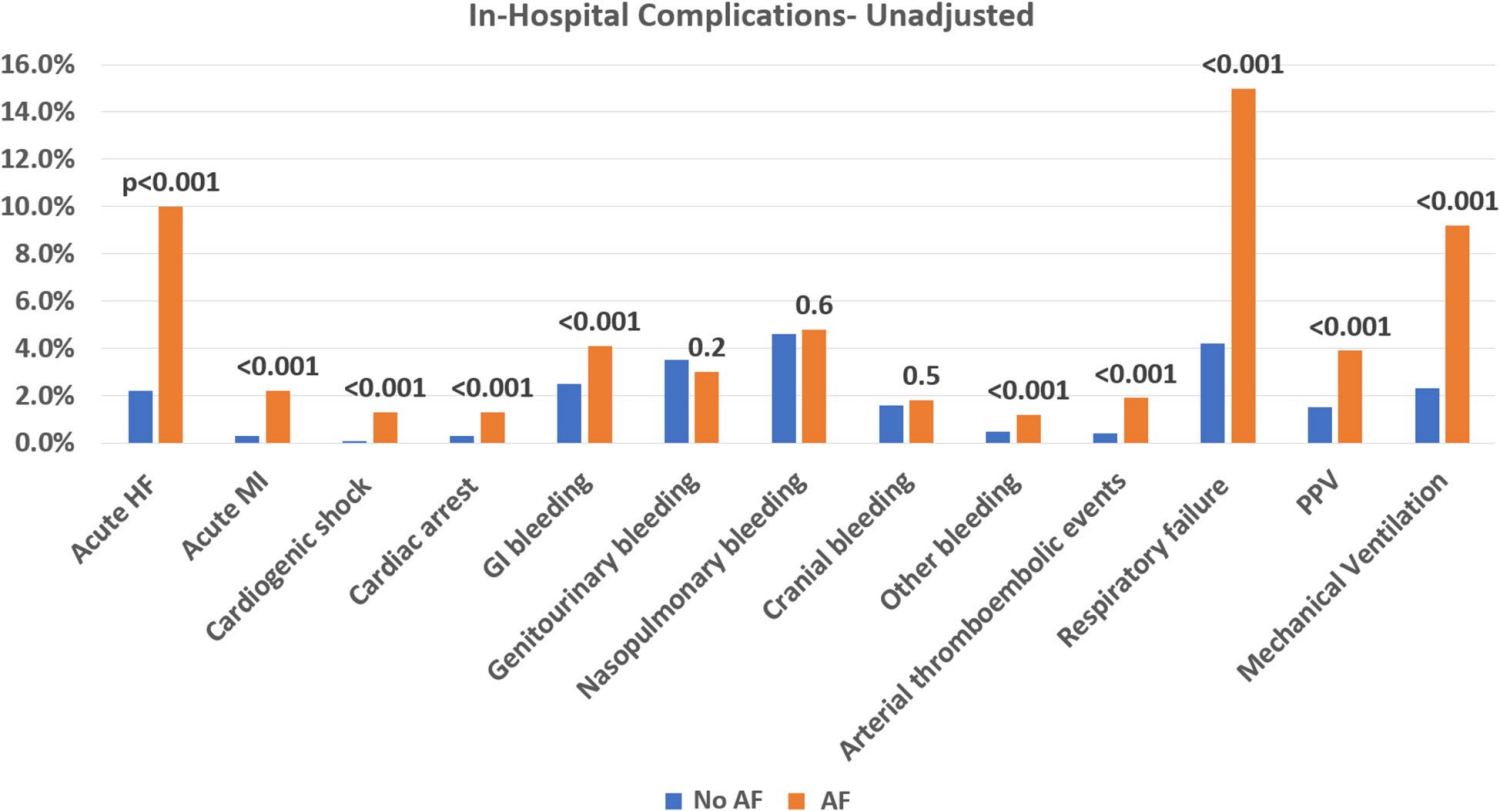
Unadjusted in hospital complications. HF: heart failure; MI: myocardial infarction; GI: gastrointestinal; PPV: positive pressure ventilation; AF: atrial fibrillation/atrial flutter

**Fig. 3:**
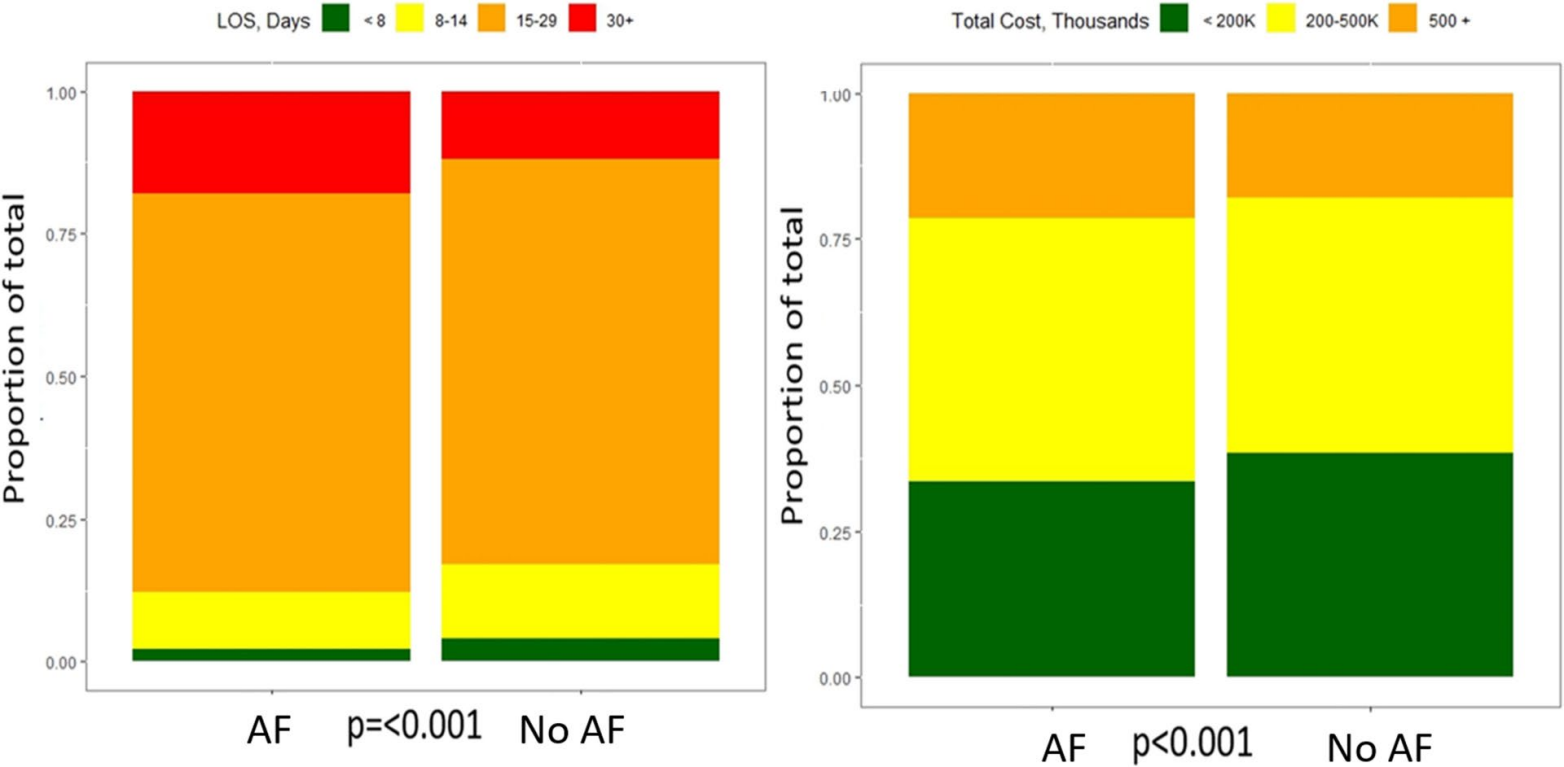
Length and cost of hospitalization for SCT. LOS: length of stay; AF: atrial fibrillation/atrial flutter

Out of the entire cohort, 1456 (2.5%) patients died during the admission for SCT. In-hospital mortality was about fourfold higher in patients with AF compared to those without AF (415 [7.2%] vs. 1041 [1.9%] deaths, *p* < 0.001). In unadjusted analysis, patients with AF had higher incidence of acute heart failure, acute myocardial infarction, cardiogenic shock, cardiac arrest, bleeding complications, thromboembolic events, and acute respiratory failure requiring ventilatory support compared to patients without AF (Fig. 2). They also had increased risk of acute kidney injury (1665 [29%] vs. 7281 [14%] events, *p* < 0.001). Additionally, the AF group had longer hospitalization (median of 21 [IQR 16,27] vs. 19 [IQR 15,25] days, *p* < 0.001) with higher cost (median of $268,031 [IQR $170,957, $455739] vs. $250,178 [IQR $153,680, $415239], *p* < 0.001) compared to the no-AF group. Specifically, more patients with AF stayed over 30 days in the hospital and had a hospitalization cost of > 500 thousand US Dollars compared to those without AF (Fig. 3).

After adjusting for several differences in baseline demographics, comorbidities (using the Charlson Comorbidity Index), indication for SCT, and type of SCT, patients with AF had higher in-hospital mortality (adjusted OR 3.65 [95% CI 3.02–4.41]) and in-hospital adverse events including cardiac complications (adjusted OR 4.92 [4.22–5.75]), bleeding (adjusted OR 1.32 [1.15–1.53]), and respiratory failure (adjusted OR 3.40 [2.97–3.90]) as compared to patients without AF (Table 2). Also, the AF group had longer adjusted hospitalization (Incidence rate ratio of 1.12 [1.1–1.15], *p* < 0.001). The individual effects of variables in the adjustment model on outcomes during IA are shown in supplemental Table 3.

In sensitivity analyses, the AF group continued to be at significantly increased risk for inpatient mortality after excluding patients with sepsis (adjusted OR 2.99 [1.91–4.69], *p* < 0.001) and after excluding patients with respiratory failure (adjusted OR 2.35 [1.16–4.77], *p* < 0.001).

### Outcomes after hospital discharge

**Fig. 4:**
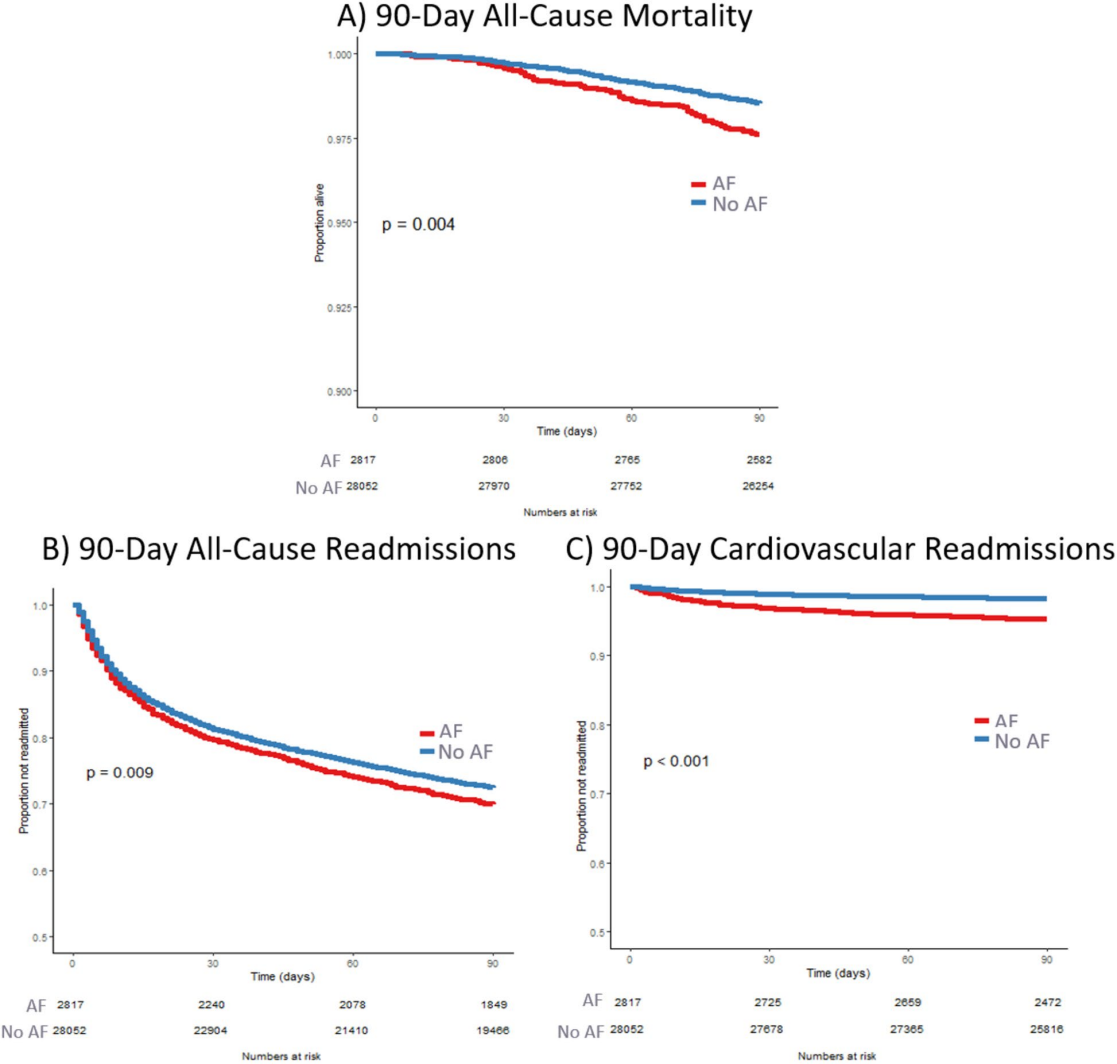
90-day mortality and readmissions. AF: atrial fibrillation/atrial flutter

Including deaths during IA, patients with AF had higher 90-day all-cause inpatient mortality than those without AF (542 [9.4%] vs. 1819 [3.4%] deaths, *p* < 0.001) respectively. Among 57,828 patients discharged alive after IA, those with AF had significantly higher 90-day all-cause inpatient mortality compared to those without AF (2.4% vs. 1.4% died, *p* = 0.001) (Fig. 4A).

Patients in the AF group were also more likely to be readmitted within 90 days and had higher all-cause readmission rates over time compared to those in the no-AF group (30% vs. 28% readmitted at 90 days, *p* = 0.02) and (171.7 vs. 138.6 readmissions per 1000 patient-follow-up months, relative risk (RR) 1.24 [1.15–1.33]) (Fig. 4B). Similarly, patients with AF were more likely to be readmitted for a primary CV cause within 90 days compared to their counterparts without AF (4.7% vs. 1.8%, *p* < 0.001) (Fig. 4C).

Patients with AF had higher adjusted 90-day all-cause inpatient mortality (Adjusted Hazard Ratio (HR) 1.54 [1.19–1.99], *p* = 0.001), all-cause readmissions (Adjusted HR 1.15 [1.07–1.24], p < 0.001), and CV readmissions (Adjusted HR 2.29 [1.85–2.82], p < 0.001) after accounting for differences in baseline demographics, comorbidities (using the Charlson Comorbidity Index), indication and type of SCT. The observed differences in mortality and readmissions between both groups persisted at 180-day follow-up (adjusted HR for mortality AF vs. no- AF 1.41 [1.17–1.70] and adjusted HR for readmission AF vs. no AF 1.18 [1.10–1.27]) (Supplemental Fig. 1). The individual effects of variables included in the adjustment model on outcomes during follow-up is shown in supplemental Table 4. The results were unchanged when excluding patients with sepsis or respiratory failure during IA for SCT (Supplemental Fig. 2).

**Fig. 5:**
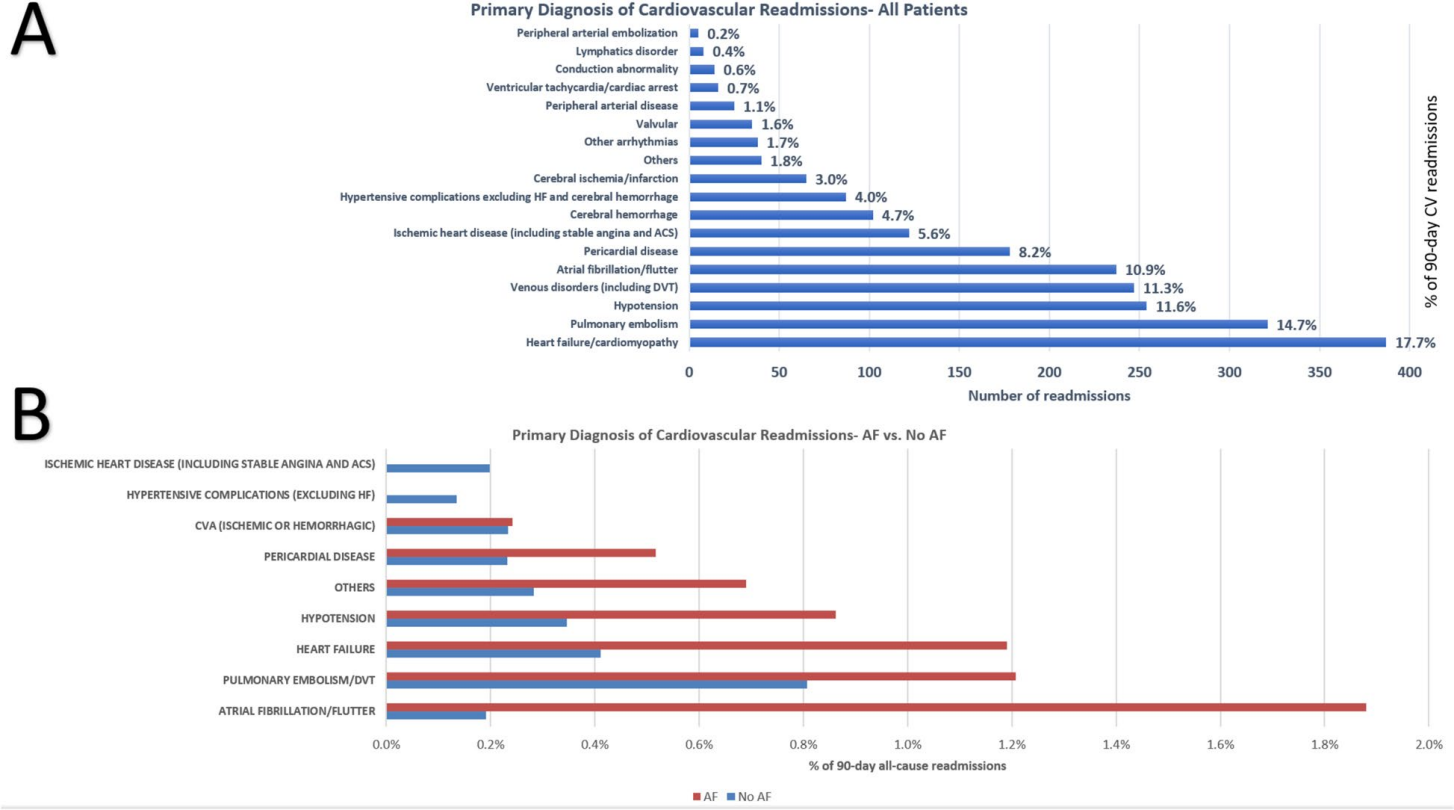
90-day cardiovascular readmissions. Panel A illustrates a breakdown of 90-day cardiovascular readmissions in all patients receiving stem cell transplantation. The y-axis shows the primary cardiovascular readmission diagnosis, the x-axis shows the number of readmissions for each diagnosis, the % next to each bar shows the proportion of each primary diagnosis out of all 90-day cardiovascular readmissions. Panel B illustrates a comparison of 90-day cardiovascular readmissions between patients with vs. without AF, the y-axis shows the primary cardiovascular readmission diagnosis, the x-axis shows the proportion of of each cardiovascular diagnosis out of 90-day all-cause readmissions. ACS: acute coronary syndrome; DVT: deep vein thrombosis; AF: atrial fibrillation/atrial flutter; HF: heart failure; CVA: cerebrovascular accident

CV readmissions accounted for 2181 (5.5% of total) readmissions, among which heart failure/cardiomyopathy was the most common cause (Fig. 5A). Figure 5B illustrates a comparison between the primary diagnosis of CV readmissions between both groups. AF were the most common primary cause of CV readmissions among the AF group (1.9% of all-cause 90-day readmissions) while venous thromboembolic events (pulmonary embolism and deep vein thrombosis) were the most common primary cause of CV readmissions in the no-AF group (0.8% of all-cause 90-day readmissions).

Among patients with AF during IA for SCT, 2.3% were readmitted for a primary diagnosis of AF during the same calendar year, whereas in patients without AF during IA for SCT, 1.8% were readmitted with a new diagnosis of AF in any diagnostic position. Patients in the AF group had more arterial thromboembolic events during calendar-year follow-up compared to those without AF (1.2% vs. 0.6%, *p* = 0.002) respectively. Most of those thromboembolic events (84%) were related to the central nervous system (such as thromboembolic strokes and transient ischemic attacks), while 16% were peripheral thromboembolic events (such as central retinal artery occlusion, intestinal infarction, embolization to the extremities). There was no difference in bleeding events between both groups (7.4% vs. 6.8%, *p* = 0.29).

## Discussion

In a retrospective analysis of over 59 thousand patients hospitalized for SCT across the US, we report several important findings. 1) AF are common among SCT recipients with a prevalence higher than the general population [16]. 2) During the hospitilization for SCT, AF were associated with increased in-hospital mortality and risk of CV, respiratory, and bleeding complications. 3) AF at the time of SCT were also associated with increased 90-day inpatient mortality, all-cause readmissions, and CV readmissions. 4) Patients with AF had longer and more expensive index hospitilizations along with higher readmission rates contributing to an increased financial burden. 5) Patients with AF were generally older with increased burden of comorbidities, though AF were independendly associated with all of the observed adverse outcomes even after adjusting for several potential baseline confounders. To our knowledge, our study is the first to investigate the association between AF and post-discharge outcomes in a large administrative database that represents real-world data across the US.

We employed several rigorous methodological and statistical approaches to leverage the large NRD dataset and reduce risk of confounding and inherent limitations of retrospective data. First, we identified the index admission for SCT, baseline characteristics, and the presence or absence of AF using preidentified ICD-10 diagnostic and procedural codes that have been used in prior literature. We also defined our outcomes and grouped them into diagnostic groups using previously published codes of claims. We included patients between January and September of each year to allow for at least 90-day follow up of our primary outcomes (mortality and readmissions). Then, to reduce risk of confounding we constructed multivariable regression models to adjust all outcomes for differences in baseline medical comorbidities and sociodemographic characteristics. By using the previously validated and widely utilized Charlson Comorbidity Index, we were able to account for differences in over 17 baseline conditions that are known to affect outcomes in administrative research. We also performed sensitivity analyses to rule out possible confounding by sepsis and respiratory failure. Finally, during follow up, we identified the cause of readmissions using the primary diagnostic code of claim to increase specificity and reduce repetitive coding (i.e. a previous event reported again during a subsequent hospitalization).

The association between AF and SCT has been long recognized. In a prior report of 395 multiple myeloma patients undergoing SCT between 2002 and 2005 at the Mayo Clinic, 10% of patients developed new AF, mostly in the first month after SCT [17]. Another study by Sureddi et al. reported a higher incidence rate of AF where 27% of 278 multiple myeloma patients developed new AF at a mean of 14.8 days following SCT [18]. Several pathways may help explain the link between AF and SCT. i) Early studies that investigated the role of intracardiac stem cells in cardiac regeneration following myocardial infarction raised concerns for proarrhythmic properties of those cells by enhancing re-entry, automaticity, and changing the properties of cardiac ion channels. Thus it is possible that peripherally injected stem cells could also be arrhythmogenic [19]. ii) Several chemotherapies such as anthracyclines, and conditioning regimens such as melphalan and busulfan used before and around the time of SCT are associated with AF [20]. iii) Acute stressors commonly encountered around SCT such as sympathetic surges, inflammation, febrile neutropenia, sepsis, pain, and dehydration are known triggers of AF. iv) Rapid fluid shifts after SCT were linked with weight gain and diastolic dysfunction, both found to be synergistic predictors of developing AF [17]. v) There is also a relatively high prevalence of traditional CV risk factors that are well known to increase the risk of AF among cancer patients receiving SCT [8].

The prevalence of AF at the time of SCT in our report (9.8%) was similar to several prior cohorts reporting a prevalence of around 9–12% [15, 17, 21, 22]. Our findings of increased mortality and adverse events in patients with AF both during and after hospitalization for SCT is also consistent with prior studies. An early study by Peres et al. reported an increased risk of hemodynamic compromise and mechanical ventilation in 25 patients (9% of 278 patients) who developed AF following reduced-intensity conditioning SCT [21]. Those findings are similar to our observed increased risk of cardiogenic shock, respiratory failure, and mechanical ventilation in the AF group. The same study also reported high recurrence rate of AF after discharge (76%), and high mortality rate of 40% at 100-day in those with a recurrence. This higher observed mortality rate compared to our study and other reports might be attributed to an older, more debilitated population who were selected to undergo a reduced-intensity conditioning regimen. In a larger study of 1177 consecutive patients over the age of 40 receiving SCT, patients with AF had ninefold higher in-hospital mortality compared to those without [22]. However, the relatively small sample size and event rate in both studies limited their ability to adjust for baseline differences in CV risk factors and prior events that could confound the observations. By leveraging the large multicenter national data in the NRD, we were able to statistically account for over 17 baseline parameters calculated through the Charlson Comorbidity Index [12], in addition to other potential confounding factors such as gender, primary payer, type, and indication for SCT. We observed an adjusted 3.7-fold increase in in-hospital mortality, a 4.9-fold increase in CV adverse events, and a 3.4-fold increase in respiratory failure in patients with AF. Our findings are also consistent with recent large studies that used the National Inpatient Sample (NIS) Database [15, 23]. Both studies adjusted for baseline sociodemographic and CV risk differences and reported around a threefold increase in in-hospital mortality. They also found a similar increase in cardiac and respiratory complications including cardiogenic shock, acute heart failure, cardiac arrest, and mechanical ventilation. These studies, though, were not able to evaluate for post-discharge outcomes as we were able to with the National Readmissions Database. Collectively, these findings may suggest an association between AF and adverse outcomes during the admission for SCT, this association appears to be independent of age and baseline differences in CV risk factors that are more prevalent in patients with AF.

AF may also pose increased financial burden on patients and healthcare systems. In our study, patients in the AF group had significantly longer hospitalizations and higher hospitalizations cost compared to their counterparts without AF. This was mostly notable among patients who required long (> 30 days) and expensive (> $500,000) hospitalizations. While our data lacks the granularity to further explore and explain these differences, it is possible that the increased incidence of CV complications, hemodynamic compromise, and respiratory failure resulted in more utilization of intensive care units (ICUs) resources prolonging the length of stay and increasing costs. Previously published studies associated AF with longer hospitalizations and increased admission to the ICU, supporting our findings and postulation [15, 22].

The association between AF and post-discharge outcomes following SCT is minimally studied. While most patients convert back to normal rhythm before discharge, the recurrence rate of AF remains unknown. In a small study, all 25 patients with AF converted to sinus rhythm by discharge, but 75% had a clinical recurrence within the first 100 days [21]. Another similarly sized study reported a clinical recurrence rate of 47% at 1-year follow-up [24]. The subclinical recurrence rate remains unknown and is probably underestimated. Prior smaller studies have also linked AF before, during, and after SCT with worse outcomes and increased mortality in patients who survive the initial hospitalization. In the study by Tonorezos et al. investigating 1177 patients undergoing SCT, 61 patients developed new onset AF following transplant and had a 3.5 fold higher 1-year mortality compared to patients who never had an AF, after adjusting for differences in age, pre-transplant arrythmias, and transplant related variables [22]. In a more recent cohort of 487 adults with no history of AF undergoing SCT, Chang and colleagues observed a significantly increased mortality among the 50 patients who developed AF during a median follow-up of 3.1 years compared to those who remained free of AF [9]. Using the pre-SCT CHARGE-AF score [25] to account for baseline differences in age, race, height, weight, blood pressure, current smoking, use of antihypertensive medication, diabetes, and history of CV disease, they observed a significantly higher all-cause and non-relapse related mortality in the AF group (adjusted HR of 12.8 and 15.8 respectively) [9]. Interestingly, in a prior study of 804 patients from our institute who underwent allogenic SCT and survived for > 1 year, pre-existing arrhythmias before SCT did not predict late adverse cardiac events, however, new onset AF was the most common late adverse cardiac event, and was the strongest predictor of late mortality (HR 10.6, 95% CI 7.7–14.6) [26].

Those prior studies have been mostly of smaller sample size and reflect individualized outcomes and practice patterns of single-centered transplant programs. Utilizing the National Readmissions Database, we were able to evaluate 59,284 weighted admissions, equivalent to 31,663 unweighted admissions. In our cohort, we similarly observed around a threefold increase in 90-day mortality in patients with AF at the time of SCT. Excluding deaths during IA, we observed higher mortality in the AF group both at 90 and 180-day follow up (adjusted HR of 1.54 [1.19–1.99] and 1.41 [1.17–1.70] respectively). By leveraging the large sample size of our cohort, we were able to show an independent association between AF and mortality even after statistically adjusting for several baseline potential confounders in CV risk factors and sociodemographic parameters. We also found in sensitivity analyses that patient with AF continued to have an over twofold increased risk of inpatient mortality even after excluding patients with sepsis and respiratory failure.

Our study is also the first large-scale analysis to investigate the effect of AF on readmissions following SCT. The presence of AF was associated with increased adjusted 90 and 180-day all-cause readmission rates and a 2.3-fold increase in 90-day CV readmissions in the AF group. While the most common causes of readmissions in both groups were related to complications of SCT and infections, CV diagnoses were a significant cause of readmission. Readmissions following SCT result in significant cost to the healthcare system, primary payers, and patients. They are also considered as a quality indicator of the performance of a hospital or a transplant program. Thus, our work may guide future efforts to improve collaboration between oncologists and cardiologists to reduce and prevent some of those readmissions. For instance, recurrent AF and acute HF were the two most common causes of readmissions in the AF group, early evaluation by cardio-oncologists or cardiologists with expertise in managing such patients could lead to implementation of outpatient strategies for volume management, HF guideline directed medical therapy (GDMT), rate, or rhythm control that may prevent some of those readmissions.

The best practices in managing AF in SCT patients remain controversial. Those patients were mostly excluded from major clinical trials and expert consensus guidelines [16]. AF around the time of SCT is associated with several complexities and clinical difficulties. First, managing the hemodynamic effects of AF are challenging, especially in patients with symptoms and/or rapid ventricular response. Those patients are often hypotensive and may also be in sepsis and/or neutropenic fever, limiting the ability to use adequate rate controlling agents like beta blockers and calcium channel blockers. Additionally, antiarrhythmic drugs that are often used for rate and rhythm control may have limitations due to impaired renal function and pharmacologic interactions with antineoplastic agents used around SCT. This may lead to a clinical situation where less preferred drugs like amiodarone or digoxin are the only feasible options. Unfortunately, in addition to its myriad of possible side effects, amiodarone may also cause hepatotoxicity in patients already at increased risk for sinusoidal obstruction syndrome or liver graft vs. host disease (GVHD) following SCT. Antiarrhythmic agents are also generally contraindicated in a thrombocytopenic patient who cannot be anticoagulated. Second, traditional risk stratification tools to predict thrombo-embolic and bleeding events like the CHA2DS2-VASc [27] and the HAS-BLED [28] scoring systems did not include cancer patients. Patients undergoing SCT often have significant thrombocytopenia that may preclude the use of anticoagulants to reduce the embolic risk. Conversely, these patients may actually be at an increased thromboembolic risk from their malignancy and hyperinflammatory state. Currently, the risk of thromboembolic and bleeding events in SCT with AF remain unknown. In the same cohort by Chang et al., 10% (5 out of 50 patients) with AF developed a clinically relevant stroke during 34.8 person-years of follow-up after AF onset. The incidence rate of stroke after AF was 143 per 1000 person-years, a rate that exceeds the highest risk class of the CHA2DS2-VASc system. However, our short-term follow-up, reliance on administrative data adjudication, and lack of data regarding the use of anticoagulation limits further interpretation or drawing conclusions. Consequently, there is a need for further study to delineate the best therapeutic strategies to guide symptom control, reduce readmissions and mortality, and balance the risk of bleeding and thromboembolic events in this challenging population.

Our results should be interpreted in the context of some important limitations. This is a retrospective analysis of an administrative dataset that uses ICD-10 codes to capture diagnoses and procedure, this carries inherent risk for confounding and diagnostic misclassification. However, we used codes in previously utilized in published similar studies [15] and employed various models to adjust for possible confounders to reduce these risks. Additionally, the ICD-10 diagnostic codes of AF and the nature of the NRD does not allow differentiating new onset from preexisting AF at the time of SCT. Nevertheless, previously published studied reported a pre-existing prevalence of AF among the SCT population to be around 2–6%, with most encountered AF being new-onset following SCT [9, 22, 29]. In addition the NRD database does not have diagnostic timestamps or medications data, thus the temporal relation between de-novel AF and SCT and conditioning regimens could not be studied. Also, the database only captures inpatient diagnoses and events, thus, outpatient diagnoses, events, and mortality could be missed. Furthermore, we do not have data for race, CV or cancer related medications, laboratory work, or imaging studies that could affect the results. Moreover, we only captured the primary diagnosis as the cause of readmission, this offers more confidence in the specificity of the cause of readmission but reduces the sensitivity to capture events. Also, the NRD does not allow the follow-up of an individual patient after the end of a calendar year, which limited the duration of available follow up. We did exclude patients with admissions from October to December, so that all patients had at least 90 days of follow-up. Lastly, we do not have data regarding the cause of death which limited our ability to explain and explore mortality outcomes.

## Conclusion

In a large nationwide sample of patients hospitalized for SCT, AF at the time of SCT were independently associated with increased risk of in-hospital and 90-day mortality, readmissions, and adverse CV events after accounting for age and baseline comorbidities. Our study is the first to investigate the association of AF with post-discharge outcomes, readmissions, and healthcare cost in a large nationwide sample representative of real-world data. Awareness of this association is important to identify a group of patients at a higher risk for mortality and adverse events to implement early preventative strategies to reduce risk and improve outcomes. Optimal management strategies for AF in patients with cancer and particularly SCT recipients is still unknown; future clinical trials and prospective studies are needed to guide the best approach in managing symptoms, reducing clinical events, and balancing the risk of bleeding and thrombo-embolism in a challenging population that is at increased risk for both.

## Supplementary Information

Supplementary Material 1

Supplementary Material 2: Supplemental Figure 1. 180-day mortality and readmissions. AF: atrial fibrillation/atrial flutter.

Supplementary Material 3: Supplemental Figure 2. 90-day mortality and readmissions excluding patients with respiratory failure and sepsis. AF: atrial fibrillation/atrial flutter

## Data Availability

The datasets generated and/or analyzed during the current study are available in the [HEALTHCARE COST & UTILIZATION PROJECT (HCUP)] repository, https://hcup-us.ahrq.gov/db/nation/nrd/nrddbdocumentation.jsp.

## Abbreviations

AF: Atrial fibrillation or atrial flutter
ALL: Acute lymphocytic leukemia
AML: Acute myeloid leukemia
CV: Cardiovascular
CVA: Cerebrovascular accident
GDMT: Guideline directed medical therapy
HCUP: The Healthcare Cost and Utilization Project
HF: Heart failure
HR: Hazard ratio
IA: Index admission
ICD-10: International Classification of Diseases 10th Edition
ICD-10-PCS: International Classification of Diseases 10th Edition Procedural Coding System
ICU: Intensive care unit
IQR: Interquartile range
MDS: Myelodysplastic syndrome
MI: Myocardial infarction
NIS: National Inpatient Sample Database
NRD: The United States National Readmission Database
OR: Odds ratio
SCT: Stem cell transplantation
SD: Standard deviation
