# Supplementary material for: Atrial fibrillation or flutter in patients undergoing stem cell transplantation, in-hospital and post-discharge outcomes in a large nationwide sample across the United States
Source: Cardiooncology. 2025 Jul 3;11:61. doi: 10.1186/s40959-025-00346-1 (PMC12225381; doi:10.1186/s40959-025-00346-1)
Supplement: Supplementary file 1 — Supplementary Material 1 [file 40959_2025_346_MOESM1_ESM.docx]

Supplemental Table 1: Diagnostic and procedural codes for baseline characteristics.

| Baseline Characteristic | Source | Diagnostic Code | Procedural Code | |
| --- | --- | --- | --- | --- |
| Atrial Fibrillation/Atrial Flutter | ICD-10 | I48 |  | |
| Type of SCT   - Autologous HSCT - Allogenic HSCT - Autologous BMT - Allogenic BMT - Autologous Cord - Allogenic Cord | ICD-10 |  | | 30230Y0, 30233Y0, 30240Y0, 30243Y0, 30250Y0, 30253Y0, 30260Y0, 30263Y0,  30230Y2, 30230Y3,  30230Y4, 30233Y2, 30233Y3, 30233Y4, 30240Y2, 30240Y3, 30240Y4,30243Y2,  30243Y3, 30243Y4, 30250Y1, 30253Y1, 30260Y1, 30263Y1,  30230G0, 30233G0, 30240G0, 30243G0, 30250G0, 30253G0, 30263G0, 30263G0  30230G2. 30230G3,  30230G4, 30233G2,  30233G3, 30233G4,  30240G2, 30240G3,  30240G4, 30243G2,  30243G3, 30243G4,  30250G1, 30253G1,  30260G1, 30263G1  30230X0, 30233X0, 30240X0, 30243X0, 30250X0, 0253X0, 30260X0, 30263X0  30230X2, 30230X3, 30230X4,  30233X2, 30233X3, 30233X4, 30240X2, 30240X3, 30240X4, 30243X2,  30243X3, 30243X4, 30250X1, 30253X1,  30260X1, 30263X1 |
| Age, years (SD) | NRD File |  |  | |
| Gender | NRD File |  |  | |
| Primary Payer | NRD File |  |  | |
| Zip code income quartile | NRD File |  |  | |
| Hospital type and size | NRD File |  |  | |
| Hypertension | ICD-10 | I10, I11, I12, I13, I14, I15, I16 |  | |
| Congestive Heart Failure | ICD-10 | I110, I130, I132, I5022, I5032, I5042 |  | |
| Coronary artery disease | ICD-10 | I20, I21, I22, I23, I24, I25 |  | |
| CABG | ICD-10 | I2570, I2571, I2572, I2573, I2576, I2579, I25810, I25812, T8221, Z951 |  | |
| Diabetes | ICD-10 | E08, E09, E10, E11, E13 |  | |
| Peripheral vascular disease | ICD-10 | E085, E095, E105, E115, E135, I73, T82856, Z9862, Z95820 |  | |
| CKD stage 3 or more | ICD-10 | N18.3, N18.4, N18.5, N18.6, E08.2, E09.2, E10.2, E11.2, E13.2, I12, I13 |  | |
| Obesity | ICD-10 | E66, Z68.3, Z68.4 |  | |
| Prior stroke/TIA | ICD-10 | I693, Z8673 |  | |
| Hyperthyroidism | ICD-10 | E05 |  | |
| Alcohol use disorder | ICD-10 | F10, Z7140, K29.2, G312, K852, K860, T51, I426, K70, G621 |  | |
| Sleep apnea | ICD-10 | G473 |  | |
| COPD | ICD-10 | J41, J42, J43, J44 |  | |
| Indication for SCT:   - Multiple myeloma - ALL - AML - Hodgkin’s lymphoma - Non-Hodgkin’s lymphoma - MDS - Others |  | C900, C6901, C902, C903  C910  C920, C923, C924, C925, C926, C929 C92A, C930, C940, C942  C810, C811, C812, C813, C814, C817, C819  C820, C821, C822, C823, C824, C825, C826, C828, C829, C830, C831, C833, C835, C837, C838, C839, C840, C841, C844, C846, C847, C849, C84A, C84Z, C851, C852, C858, C859, C860, C861, C862, C863, C864, C865, C866, C884  C946, D469, D46C, D46Z |  | |
| Charlson Comorbidity Index | ICD-10 calculated |  |  | |

Supplemental Table 2: diagnostic codes for events

| Outcome | Source | Diagnostic Code | Procedural Code |
| --- | --- | --- | --- |
| Mortality | NRD File |  |  |
| Readmission | NRD File |  |  |
| Acute Heart Failure | ICD-10 | I5020, I5021, I5023, I5030, I5031, I5033, I5040, I5041, I5043, I5082, I509, I5089, I5084 |  |
| Acute Myocardial Infarction | ICD-10 | I21, I22, I23 | 0270X, 0271X, 0272X, 0273X, 02C03X, 02C04X, 02C13X, 02C14X, 02C23X, 02C24X, 02C33X, 02C34X, 0210x, 0211x, 0212x, 0213x |
| Cardiogenic Shock | ICD-10 | R570 |  |
| Cardiac Arrest | ICD-10 | I462, I468, I469 |  |
| Acute Kidney Injury | ICD-10 | N17 |  |
| Acute Respiratory Failure | ICD-10 | J960, J962, J969 |  |
| Non-invasive positive pressure ventilation | ICD-10 |  | 5A0935, 5A0945, 5A0955 |
| Mechanical Ventilation | ICD-10 |  | 5A1935Z, 5A1945Z, 5A1955Z |
| Gastrointestinal Bleeding | ICD-10 | I8501, I8511, K2081, K2091, K2101, K2211, K2901, K2921, K2931, K2941, K2951, K2961, K2971, K2981, K2991, K31811, K50011, K50111, K50811, K50911, K51011, K51211, K51311, K51411, K51511, K51811, K51911, K5701, K5711, K5713, K5721, K5731, K5733, K5741, K5751, K5753, K5781, K5791, K5793, K920, K921, K226, K250, K252, K260, K262, K270, K272, K280, K282, K3182, K5521, K625, K922, K9401, K9411, K9421, K9431 |  |
| Genitourinary Bleeding | ICD-10 | N923, N924, N930, N938, N939, N950, N02-, N3001, N3011, N3021, N3031, N3041, N3081, N3091, R310, R319, N421, N99510, N99520, N99530 |  |
| Nasopulmonary Bleeding | ICD-10 | R040, R042, R0489, R049 |  |
| Intracranial Bleeding | ICD-10 | G0430, G0439, G361, H0523, H113, H313, H314, H356, H357, H431, H4702, H6032, I60, I61, I62, S063, S064, S065, S066 |  |
| Other/Unspecified Bleeding | ICD-10 | D62, D698, D699, R58, T792XXA |  |
| Arterial Thromboembolism | ICD-10 | G9511, I630, I631, I632, I633, I634, I635, I638, I639, I65, I66, I741, I742, I743, I744, I745, I748, I749, I750-, I758, G459, G460, G461, G462, G463, G464, G465, G466, G467, H340, H341, H342, K550 |  |
| Sepsis | ICD-10 | A021, A207, A227, A267, A327, A40x, A41x, A427, A5486, B377, R6520, R6521, R7881, T8112, T8144 |  |

Supplemental Table 3: adjustment models for outcomes during index admission

| **Variable** | **Label** | **Estimate** | **95% CI** | **p-value** |
| --- | --- | --- | --- | --- |
| **In-hospital Mortality** | | | | |
| AF | AF | 3.65 | 3.02, 4.41 | <0.0001 |
| AGE | AGE | 1.00 | 0.99, 1.01 | 0.7983 |
| Sex (Ref: Female) | Male | 0.82 | 0.69, 0.96 | 0.0162 |
| Charlson Comorbidity Index | Charlson | 1.35 | 1.29, 1.40 | <0.0001 |
| PRIM_INS1 (Ref: Medicare) |  |  |  |  |
|  | Medicaid | 0.91 | 0.69, 1.20 | 0.5067 |
|  | Other | 0.70 | 0.47, 1.06 | 0.0907 |
|  | Private | 0.73 | 0.61, 0.89 | 0.0013 |
|  |  |  |  |  |
| Type of SCT (ref: Allogenic) | Autologous | 0.22 | 0.15, 0.32 | <0.0001 |
|  | |  |  |  |
| Source of SCT (ref: Bone marrow) | Cord | 3.09 | 1.89, 5.05 | <0.0001 |
|  | Peripheral SCT | 1.55 | 1.04, 2.30 | 0.0300 |
| Indication for SCT (ref: AML) |  |  |  |  |
|  | ALL | 1.27 | 0.93, 1.74 | 0.1327 |
|  | Hodgkin lymphoma | 1.24 | 0.70, 2.18 | 0.4550 |
|  | MDS | 1.75 | 1.32, 2.33 | 0.0001 |
|  | Multiple myeloma | 0.62 | 0.39, 1.00 | 0.0477 |
|  | Non-Hodgkin lymphoma | 1.47 | 1.03, 2.10 | 0.0359 |
|  | Other | 1.64 | 1.23, 2.19 | 0.0008 |
| **Bleeding Complications** | | | | |
| AF | AF | 1.32 | 1.15, 1.53 | 0.0001 |
| AGE | AGE | 0.99 | 0.98, 0.99 | 0.0000 |
| Sex (Ref: Female) | Male | 0.95 | 0.87, 1.02 | 0.1703 |
| Charlson Comorbidity Index | Charlson | 1.08 | 1.05, 1.12 | <0.0001 |
| PRIM_INS1 (Ref: Medicare) |  |  |  |  |
|  | Medicaid | 0.92 | 0.78, 1.08 | 0.2852 |
|  | Other | 0.74 | 0.60, 0.92 | 0.0059 |
|  | Private | 0.89 | 0.80, 0.98 | 0.0198 |
|  |  |  |  |  |
| Type of SCT (ref: Allogenic) | Autologous | 0.55 | 0.48, 0.63 | <0.0001 |
|  |  |  |  |  |
| Source of SCT (ref: Bone marrow) | Cord | 1.08 | 0.81, 1.45 | 0.5972 |
|  | Peripheral SCT | 0.95 | 0.80, 1.12 | 0.5158 |
| Indication for SCT (ref: AML) |  |  |  |  |
|  | ALL | 1.19 | 1.01, 1.40 | 0.0335 |
|  | Hodgkin lymphoma | 0.96 | 0.79, 1.18 | 0.7278 |
|  | MDS | 1.29 | 1.08, 1.54 | 0.0048 |
|  | Multiple myeloma | 0.51 | 0.43, 0.61 | <0.0001 |
|  | Non-Hodgkin lymphoma | 1.07 | 0.93, 1.24 | 0.3341 |
|  | Other | 1.32 | 1.14, 1.53 | 0.0002 |
| Respiratory Complications | | | | |
| AF | AF | 3.40 | 2.97, 3.90 | <0.0001 |
| AGE | AGE | 1.01 | 1.00, 1.01 | 0.0158 |
| Sex (Ref: Female) | Male | 0.91 | 0.82, 1.01 | 0.0753 |
| Charlson Comorbidity Index | Charlson | 1.27 | 1.24, 1.31 | 0.0000 |
| PRIM_INS1 (Ref: Medicare) |  |  |  |  |
|  | Medicaid | 0.92 | 0.75, 1.14 | 0.4617 |
|  | Other | 0.89 | 0.71, 1.13 | 0.3419 |
|  | Private | 0.79 | 0.70, 0.91 | 0.0007 |
|  |  |  |  |  |
| Type of SCT (ref: Allogenic) | Autologous | 0.39 | 0.31, 0.49 | <0.0001 |
|  |  |  |  |  |
| Source of SCT (ref: Bone marrow) | Cord | 2.18 | 1.30, 3.67 | 0.0034 |
|  | Peripheral SCT | 1.22 | 0.93, 1.60 | 0.1582 |
| Indication for SCT (ref: AML) |  |  |  |  |
|  | ALL | 1.24 | 0.99, 1.56 | 0.0621 |
|  | Hodgkin lymphoma | 1.14 | 0.80, 1.64 | 0.4704 |
|  | MDS | 1.84 | 1.44, 2.37 | <0.0001 |
|  | Multiple myeloma | 0.91 | 0.68, 1.21 | 0.5126 |
|  | Non-Hodgkin lymphoma | 1.31 | 1.03, 1.68 | 0.0300 |
|  | Other | 1.67 | 1.34, 2.08 | <0.0001 |
| **Cardiac Complications** | | | | |
| AF | AF | 4.92 | 4.22, 5.75 | <0.001 |
| AGE | AGE | 1.03 | 1.02, 1.04 | <0.001 |
| Sex (Ref: Female) | Male | 0.81 | 0.71, 0.93 | 0.0023 |
| Charlson Comorbidity Index | Charlson | 1.48 | 1.43, 1.52 | <0.001 |
| PRIM_INS1 (Ref: Medicare) |  |  |  | 0.6620 |
|  | Medicaid | 1.20 | 0.88, 1.64 | 0.2498 |
|  | Other | 1.09 | 0.75, 1.59 | 0.6524 |
|  | Private | 1.02 | 0.88, 1.18 | 0.8156 |
|  |  |  |  |  |
| Type of SCT (ref: Allogenic) | Autologous | 0.49 | 0.38, 0.63 | <0.001 |
|  |  |  |  | 0.8629 |
| Source of SCT (ref: Bone marrow) | Cord | 1.21 | 0.59, 2.50 | 0.6025 |
|  | Peripheral SCT | 1.05 | 0.78, 1.42 | 0.7325 |
| Indication for SCT (ref: AML) |  |  |  | <0.001 |
|  | ALL | 0.97 | 0.66, 1.42 | 0.8614 |
|  | Hodgkin lymphoma | 1.77 | 1.03, 3.02 | 0.0377 |
|  | MDS | 1.69 | 1.30, 2.20 | 0.0001 |
|  | Multiple myeloma | 0.95 | 0.69, 1.30 | 0.7327 |
|  | Non-Hodgkin lymphoma | 1.11 | 0.81, 1.52 | 0.5288 |
|  | Other | 1.72 | 1.31, 2.24 | <0.001 |

Supplemental Table 4: adjustment models for post-discharge outcomes.

| **Variable** | **Label** | **Estimate** | **95% CI** | **p-value** |
| --- | --- | --- | --- | --- |
| **90-Day Mortality** | | | | |
| AF | AF | 1.54 | 1.189, 1.989 | 0.0011 |
| AGE | AGE | 1.00 | 0.995, 1.014 | 0.3305 |
| Sex (Ref: Female) | Male | 0.84 | 0.697, 1.006 | 0.0575 |
| Charlson Comorbidity Index | Charlson | 1.17 | 1.103, 1.243 | 0.0000 |
| PRIM_INS1 (Ref: Medicare) |  |  |  |  |
|  | Medicaid | 0.96 | 0.651, 1.409 | 0.8281 |
|  | Other | 1.04 | 0.668, 1.616 | 0.8650 |
|  | Private | 0.90 | 0.721, 1.123 | 0.3518 |
|  |  |  |  |  |
| Type of SCT (ref: Allogenic) | Autologous | 0.19 | 0.140, 0.270 | 0.0000 |
|  | |  |  |  |
| Source of SCT (ref: Bone marrow) | Cord | 0.82 | 0.401, 1.692 | 0.5979 |
|  | Peripheral SCT | 0.86 | 0.583, 1.276 | 0.4600 |
| Indication for SCT (ref: AML) |  |  |  |  |
|  | ALL | 0.92 | 0.637, 1.329 | 0.6571 |
|  | Hodgkin lymphoma | 1.11 | 0.586, 2.102 | 0.7486 |
|  | MDS | 1.74 | 1.253, 2.412 | 0.0009 |
|  | Multiple myeloma | 0.63 | 0.384, 1.047 | 0.0751 |
|  | Non-Hodgkin lymphoma | 1.31 | 0.932, 1.841 | 0.1205 |
|  | Other | 0.94 | 0.674, 1.301 | 0.6956 |
| **180-Day Mortality** | | | | |
| AF | AF | 1.41 | 1.173, 1.701 | 0.0003 |
| AGE | AGE | 1.00 | 0.997, 1.010 | 0.2898 |
| Sex (Ref: Female) | Male | 0.94 | 0.814, 1.075 | 0.3482 |
| Charlson Comorbidity Index | Charlson | 1.15 | 1.101, 1.200 | 0.0000 |
| PRIM_INS1 (Ref: Medicare) |  |  |  |  |
|  | Medicaid | 1.04 | 0.810, 1.330 | 0.7672 |
|  | Other | 0.90 | 0.650, 1.236 | 0.5042 |
|  | Private | 0.86 | 0.727, 1.026 | 0.0955 |
|  |  |  |  |  |
| Type of SCT (ref: Allogenic) | Autologous | 0.27 | 0.203, 0.361 |  |
|  |  |  |  | 0.2505 |
| Source of SCT (ref: Bone marrow) | Cord | 1.50 | 0.926, 2.426 | 0.0996 |
|  | Peripheral SCT | 1.17 | 0.894, 1.535 | 0.2518 |
| Indication for SCT (ref: AML) |  |  |  |  |
|  | ALL | 1.01 | 0.789, 1.282 | 0.9634 |
|  | Hodgkin lymphoma | 0.64 | 0.376, 1.088 | 0.0993 |
|  | MDS | 1.72 | 1.387, 2.125 | 0.0000 |
|  | Multiple myeloma | 0.38 | 0.260, 0.565 | 0.0000 |
|  | Non-Hodgkin lymphoma | 0.95 | 0.713, 1.253 | 0.6955 |
|  | Other | 1.00 | 0.816, 1.234 | 0.9748 |
| **90-Day All-Cause Readmissions** | | | | |
| AF | AF | 1.15 | 1.066, 1.239 | 0.0003 |
| AGE | AGE | 0.99 | 0.991, 0.996 | 0.0000 |
| Sex (Ref: Female) | Male | 0.98 | 0.940, 1.024 | 0.3842 |
| Charlson Comorbidity Index | Charlson | 1.13 | 1.107, 1.147 | 0.0000 |
| PRIM_INS1 (Ref: Medicare) |  |  |  |  |
|  | Medicaid | 0.93 | 0.841, 1.026 | 0.1468 |
|  | Other | 1.03 | 0.906, 1.169 | 0.6591 |
|  | Private | 0.89 | 0.831, 0.956 | 0.0013 |
|  |  |  |  |  |
| Type of SCT (ref: Allogenic) | Autologous | 0.53 | 0.462, 0.608 | 0.0000 |
|  |  |  |  |  |
| Source of SCT (ref: Bone marrow) | Cord | 1.15 | 0.942, 1.398 | 0.1717 |
|  | Peripheral SCT | 1.00 | 0.898, 1.122 | 0.9460 |
| Indication for SCT (ref: AML) |  |  |  |  |
|  | ALL | 1.11 | 0.987, 1.241 | 0.0837 |
|  | Hodgkin lymphoma | 0.58 | 0.484, 0.703 | 0.0000 |
|  | MDS | 1.35 | 1.204, 1.504 | 0.0000 |
|  | Multiple myeloma | 0.64 | 0.531, 0.761 | 0.0000 |
|  | Non-Hodgkin lymphoma | 0.89 | 0.774, 1.022 | 0.0982 |
|  | Other | 1.30 | 1.167, 1.439 | 0.0000 |
| **180-Day All-Cause Readmissions** | | | | |
| AF | AF | 1.18 | 1.104, 1.272 | 0.0000 |
| AGE | AGE | 1.00 | 0.993, 0.997 | 0.0000 |
| Sex (Ref: Female) | Male | 0.98 | 0.947, 1.024 | 0.4375 |
| Charlson Comorbidity Index | Charlson | 1.12 | 1.105, 1.140 | 0.0000 |
| PRIM_INS1 (Ref: Medicare) |  |  |  |  |
|  | Medicaid | 0.98 | 0.906, 1.066 | 0.6764 |
|  | Other | 1.00 | 0.893, 1.125 | 0.9746 |
|  | Private | 0.90 | 0.850, 0.957 | 0.0006 |
|  |  |  |  |  |
| Type of SCT (ref: Allogenic) | Autologous | 0.50 | 0.445, 0.565 | 0.0000 |
|  |  |  |  |  |
| Source of SCT (ref: Bone marrow) | Cord | 1.11 | 0.919, 1.332 | 0.2869 |
|  | Peripheral SCT | 0.99 | 0.905, 1.089 | 0.8780 |
| Indication for SCT (ref: AML) |  |  |  |  |
|  | ALL | 1.08 | 0.976, 1.198 | 0.1368 |
|  | Hodgkin lymphoma | 0.59 | 0.499, 0.699 | 0.0000 |
|  | MDS | 1.35 | 1.223, 1.485 | 0.0000 |
|  | Multiple myeloma | 0.65 | 0.557, 0.769 | 0.0000 |
|  | Non-Hodgkin lymphoma | 0.91 | 0.802, 1.033 | 0.1437 |
|  | Other | 1.25 | 1.138, 1.376 | 0.0000 |
| **90-Day CV Readmissions** | | | | |
| AF | AF | 2.29 | 1.853, 2.819 | 0.0000 |
| AGE | AGE | 1.02 | 1.015, 1.031 | 0.0000 |
| Sex (Ref: Female) | Male | 0.92 | 0.789, 1.084 | 0.3349 |
| Charlson Comorbidity Index | Charlson | 1.07 | 1.014, 1.137 | 0.0154 |
| PRIM_INS1 (Ref: Medicare) |  |  |  |  |
|  | Medicaid | 0.95 | 0.674, 1.331 | 0.7559 |
|  | Other | 1.01 | 0.628, 1.610 | 0.9825 |
|  | Private | 1.01 | 0.829, 1.240 | 0.8939 |
|  |  |  |  |  |
| Type of SCT (ref: Allogenic) | Autologous | 0.56 | 0.403, 0.788 | 0.0008 |
|  |  |  |  |  |
| Source of SCT (ref: Bone marrow) | Cord | 0.72 | 0.361, 1.420 | 0.3388 |
|  | Peripheral SCT | 0.68 | 0.447, 1.022 | 0.0632 |
| Indication for SCT (ref: AML) |  |  |  |  |
|  | ALL | 0.88 | 0.592, 1.321 | 0.5495 |
|  | Hodgkin lymphoma | 0.84 | 0.421, 1.664 | 0.6111 |
|  | MDS | 0.91 | 0.628, 1.315 | 0.6102 |
|  | Multiple myeloma | 0.81 | 0.543, 1.212 | 0.3078 |
|  | Non-Hodgkin lymphoma | 0.82 | 0.556, 1.217 | 0.3279 |
|  | Other | 0.96 | 0.703, 1.316 | 0.8069 |
