# Supplementary figures and images for: Atrial fibrillation or flutter in patients undergoing stem cell transplantation, in-hospital and post-discharge outcomes in a large nationwide sample across the United States
Source: Cardiooncology. 2025 Jul 3;11:61. doi: 10.1186/s40959-025-00346-1 (PMC12225381; doi:10.1186/s40959-025-00346-1)

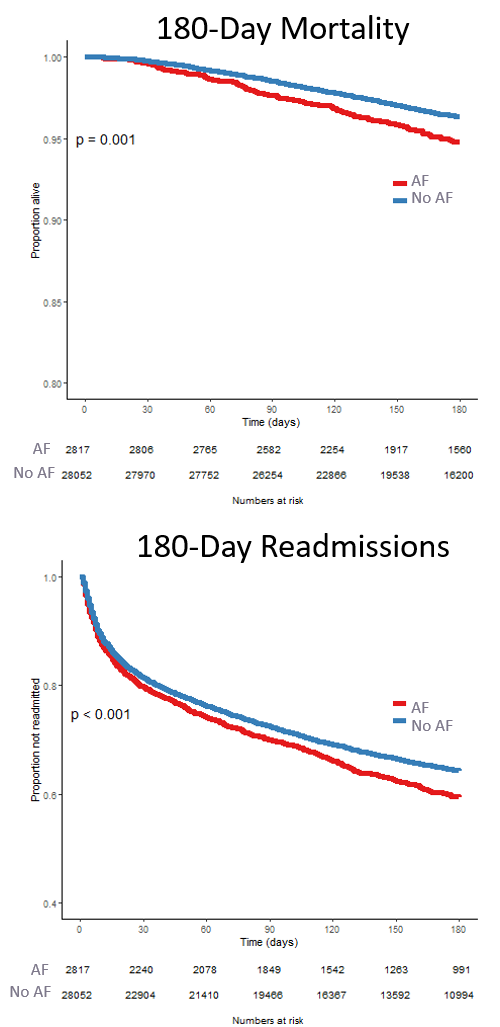

Supplement: Supplementary file 2 — Supplementary Material 2: Supplemental Figure 1. 180-day mortality and readmissions. AF: atrial fibrillation/atrial flutter. [file 40959_2025_346_MOESM2_ESM.tif]

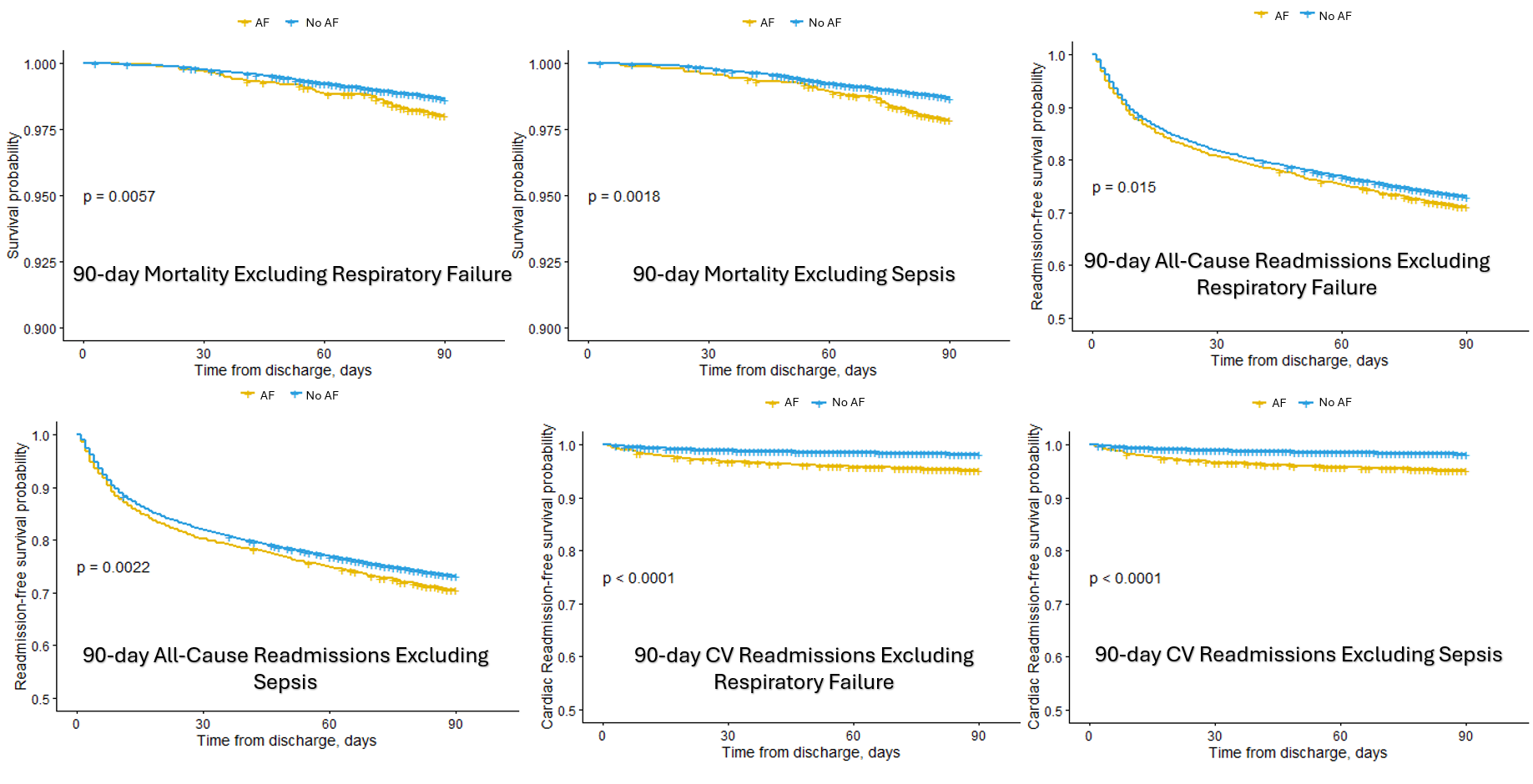

Supplement: Supplementary file 3 — Supplementary Material 3: Supplemental Figure 2. 90-day mortality and readmissions excluding patients with respiratory failure and sepsis. AF: atrial fibrillation/atrial flutter [file 40959_2025_346_MOESM3_ESM.tif]
